# Sympathetic structural and electrophysiological remodeling in a rabbit model of reperfused myocardial infarction

**DOI:** 10.1152/ajpheart.00398.2024

**Published:** 2024-07-19

**Authors:** Amanda Guevara, Charlotte E. R. Smith, Lianguo Wang, Jessica L. Caldwell, Srinivas Tapa, Samantha D. Francis Stuart, Betty W. Ma, G. Andre Ng, Beth A. Habecker, Zhen Wang, Crystal M. Ripplinger

**Affiliations:** ^1^Department of Pharmacology, University of California Davis, Davis, California, United States; ^2^Campus Veterinary Services, University of California Davis, Davis, California, United States; ^3^Department of Cardiovascular Sciences, University of Leicester, Leicester, United Kingdom; ^4^National Institute for Health & Care Research, Leicester Biomedical Research Centre, Leicester, United Kingdom; ^5^Glenfield Hospital, Leicester, United Kingdom; ^6^Department of Chemical Physiology and Biochemistry, Oregon Health and Science University, Portland, Oregon, United States; ^7^Department of Medicine and Knight Cardiovascular Institute, Oregon Health and Science University, Portland, Oregon, United States; ^8^Shantou University Medical College, Shantou, China

**Keywords:** arrhythmia, chondroitin sulfate proteoglycans, hypoinnervation, myocardial infarction, sympathetic nerve stimulation

## Abstract

Chondroitin sulfate proteoglycans (CSPGs) inhibit sympathetic reinnervation in rodent hearts post-myocardial infarction (MI), causing regional hypoinnervation that is associated with supersensitivity of β-adrenergic receptors and increased arrhythmia susceptibility. To investigate the role of CSPGs and hypoinnervation in the heart of larger mammals, we used a rabbit model of reperfused MI and tested electrophysiological responses to sympathetic nerve stimulation (SNS). Innervated hearts from MI and sham rabbits were optically mapped using voltage and Ca^2+^-sensitive dyes. SNS was performed with electrical stimulation of the spinal cord, and β-adrenergic responsiveness was tested using isoproterenol. Sympathetic nerve density and CSPG expression were evaluated using immunohistochemistry. CSPGs were robustly expressed in the infarct region of all MI hearts, and the presence of CSPGs was associated with reduced sympathetic nerve density in the infarct versus remote region. Action potential duration (APD) dispersion and tendency for induction of ventricular tachycardia/fibrillation (VT/VF) were increased with SNS in MI but not sham hearts. SNS decreased APD at 80% repolarization (APD_80_) in MI but not sham hearts, whereas isoproterenol decreased APD_80_ in both groups. Isoproterenol also shortened Ca^2+^ transient duration at 80% repolarization in both groups but to a greater extent in MI hearts. Our data suggest that sympathetic remodeling post-MI is similar between rodents and rabbits, with CSPGs associated with sympathetic hypoinnervation. Despite a reduction in sympathetic nerve density, the infarct region of MI hearts remained responsive to both physiological SNS and isoproterenol, potentially through preserved or elevated β-adrenergic responsiveness, which may underlie increased APD dispersion and tendency for VT/VF.

**NEW & NOTEWORTHY** Here, we show that CSPGs are present in the infarcts of rabbit hearts with reperfused MI, where they are associated with reduced sympathetic nerve density. Despite hypoinnervation, sympathetic responsiveness is maintained or enhanced in MI rabbit hearts, which also demonstrate increased APD dispersion and tendency for arrhythmias following sympathetic modulation. Together, this study indicates that the mechanisms of sympathetic remodeling post-MI are similar between rodents and rabbits, with hypoinnervation likely associated with enhanced β-adrenergic sensitivity.

## INTRODUCTION

Myocardial infarction (MI) is a prevailing cause of cardiovascular disease and is associated with substantial risk of ventricular arrhythmias (1, 2). In addition to myocyte loss due to ischemia following MI, there is also significant remodeling of cardiac sympathetic innervation. Post-MI sympathetic hypoinnervation is associated with worse arrhythmia outcomes and sudden cardiac death (3–5), with experimental studies indicating that nonuniform sympathetic nerve density along with changes in adrenergic sensitivity may be arrhythmogenic (6–8).

Electroanatomical mapping of hearts from human patients with MI has shown that areas of sympathetic hypoinnervation are associated with β-adrenergic supersensitivity and increased dispersion of the activation recovery interval, a surrogate for action potential (AP) duration (APD), during sympathetic stimulation (9). We have similarly shown β-adrenergic supersensitivity, Ca^2+^ mishandling, increased APD dispersion, and increased arrhythmia susceptibility in hypoinnervated post-MI mouse hearts (7), along with changes in β-adrenergic and electrophysiological responses in areas of sympathetic hypoinnervation even in the absence of ischemia or MI (10).

Previous work has suggested a role for chondroitin sulfate proteoglycans (CSPGs) in modulating cardiac innervation. In mouse models of reperfused MI, CSPGs present in the infarct inhibited sympathetic nerve reinnervation in the infarct and the adjacent myocardium (7, 11). However, mouse heart size, electrophysiology, and arrhythmia patterns differ from that of larger species (12, 13), including human, where the role of CSPG-associated hypoinnervation post-MI has yet to be explored. To address this, we used a translationally relevant rabbit model that has similar electrophysiology and arrhythmogenesis to humans (14–17) to investigate electrophysiological responses to sympathetic stimulation and determine if CSPGs and associated sympathetic hypoinnervation are present in a larger animal model of reperfused MI.

## MATERIALS AND METHODS

### Ethical Approval

All procedures involving animals were approved by the Animal Care and Use Committee of the University of California, Davis (Protocol No. 20991) and adhered to the *Guide for the Care and Use of Laboratory Animals*, published by the National Institutes of Health (NIH Publication No. 85-23, Revised 2011).

### Rabbit MI Model

New Zealand White rabbits (4–12 mo old, 2.6–3.0 kg, *N* = 22; Charles River Laboratories) were housed for ≥1 mo before the study with ad libitum access to food and water. Rabbits were randomly assigned to sham or MI surgery at a ratio of ∼1:3 to account for MI-associated mortality. For MI surgery, rabbits were sedated with butorphanol and/or acepromazine. Anesthesia was induced with ketamine and diazepam intravenously via an ear vein catheter and maintained with inhaled isoflurane (∼2%) via a ventilator for the duration of surgery. An ∼4-cm incision was made at the fourth intercostal space, and a chest retractor was placed to expose the heart and lungs. The pericardium was carefully separated to visualize the left coronary artery, and the descending branch of the left circumflex artery was ligated with a suture midway between the apex and the base for 45–60 min (18, 19). During the ligation, the retractor was removed, and the chest was closed. A bolus of lidocaine (0.5–1 mg/kg) was intravenously infused before ligation to prevent ventricular fibrillation (VF). In the event of VF, rabbits were given an additional intravenous bolus of lidocaine (0.5 mg/kg), and hearts were manually massaged by hand for recovery along with defibrillation. Sham surgeries were performed by passing the suture under the coronary artery without ligation. Heart rate (HR), body temperature, and blood pressure were monitored during surgery, with body temperature maintained at 37°C by a temperature-controlled warming pad. Ringer’s lactate solution was given via intravenous infusion during surgery to ensure adequate hydration. Once the surgery was completed, rabbits were returned to housing and monitored. Enrofloxacin (5 mg/kg) was given once to prevent infection, with buprenorphine (0.05–0.1 mg/kg) given immediately postsurgery and then twice daily for 48 h and meloxicam given once daily for 5 days. Animals remained in housing until experimental use 10 ± 1 days postsurgery.

### Sample Sizes

Of the 17 MI-operated rabbits, 10 (7 males and 3 females) survived the surgery, with the 7 deaths associated with ventricular arrhythmias during reperfusion despite lidocaine administration and defibrillation. Although more male than female animals survived, there was no sex difference in survival rate (*P* = 0.1534). Of the 10 surviving animals with MIs, 8 hearts were successfully perfused for mapping (5 males and 3 females). However, due to technical limitations of the innervated heart preparation, visualization of the infarct region within the mapping field of view and performance of all measurements or pacing protocols was not possible in every heart. As such, the number of animals is variable for some parameters and detailed within each figure legend.

### Innervated Heart Preparation

Innervated hearts were prepared as previously described (20–22). Briefly, on *day 10 ± 1* postsurgery, rabbits were administered heparin (1,000 IU), followed by an overdose of pentobarbital sodium (>100 mg/kg) intravenously via an ear vein catheter. Upon deep anesthesia, the front of the ribcage was removed, the pericardium gently pulled apart and away from the heart, and the chest filled with ice-cold cardioplegia, consisting of (in mmol/L) 110 NaCl, 1.2 CaCl_2_, 16 KCl, 16 MgCl_2_, and 10 NaHCO_3_. The descending aorta was then cannulated with an eight-gauge cannula and flushed with ice-cold cardioplegia. The heart and posterior ribcage were removed with the thoracic spinal column intact by cutting the cervical spine at C1 and the thoracic spine at T12. The preparation was then dissected from surrounding tissues and submerged into ice-cold cardioplegia. The innervated heart was moved to a glass-jacketed chamber and perfused via the descending aorta with Tyrode’s solution at 37°C, consisting of (in mmol/L) 128.2 NaCl, 1.3 CaCl_2_, 4.7 KCl, 1.05 MgCl_2_, 1.19 NaH_2_PO_4_, 20 NaHCO_3_, and 11.1 glucose. The excitation-contraction uncoupler blebbistatin (Tocris Bioscience; 10–20 µM) (23) and the nondepolarizing skeletal muscle paralytic vecuronium bromide (Cayman Chemical; 5 µM) were added to the perfusate to eliminate motion artifacts during optical recording. Perfusion pressure was maintained at 40–60 mmHg by adjusting the flow rate (∼100–120 mL/min). Three Ag/AgCl needle electrodes were positioned in the bath (2 in the thoracic cavity and 1 grounded in the chamber) for continuous ECG recording.

### Dual Optical Mapping

Dual optical mapping of intracellular Ca^2+^ and transmembrane potential (*V*_m_) was performed as previously described (13, 20, 22, 24). Briefly, hearts were loaded with 1 mg/mL Rhod-2 AM in 0.5 mL of DMSO containing 10% pluronic acid (Molecular Probes) followed by 50 µL of 1 mg/mL RH237 in DMSO (Molecular Probes). The dyes were excited at 531 ± 20 nm (LEX-2, SciMedia). Emitted fluorescence was collected using a THT-macroscope (SciMedia). Emission signals were split with a dichroic at 630 nm, long-pass filtered at 700 nm (*V*_m_), and band-pass filtered at 590 ± 16 nm (Ca^2+^). Images were acquired at 1 kHz with a 31 mm × 31 mm (100 × 100 pixels) field of view using CMOS cameras (MiCam Ultima-L, SciMedia).

### Experimental Protocol

For ventricular pacing, a bipolar electrode was placed on the surface of the mid/base right ventricle. For sympathetic nerve stimulation (SNS), a 6-Fr quadripolar catheter (2-mm electrode and 5-mm spacing) was inserted into the spinal cord to T1–T3 as previously described (20–22). As our previous studies demonstrated that rabbit hearts have a strong response to SNS in a range of 5–15 Hz and 5–10 V for no longer than 60 s as measured by changes in HR (20), hearts in this study were stimulated with SNS at 8 Hz and 10 V for 60 s in sinus rhythm or for 13 s with ventricular pacing. Rate-matched baseline electrophysiological parameters were measured during continuous ventricular pacing at a pacing cycle length (PCL) of 200 ms with or without SNS. Incidence of arrhythmia was assessed following 60 s of SNS with ventricular pacing [progressively faster PCLs in 20-ms steps until loss of capture or induction of ventricular tachycardia/fibrillation (VT/VF)]. Responsiveness to β-adrenergic stimulation was assessed with isoproterenol (Iso, 30–100 nM), which was added to the perfusate.

### Optical Mapping Data Analysis

Data analysis was conducted using Optiq (Cairn Research) and Electromap (25) as previously described (10, 22, 26). Briefly, APD (APD_80_) and Ca^2+^ transient duration (CaTD_80_) were calculated as 80% repolarization minus activation time. APD dispersion was determined by dividing the range between the 5th and 95th percentiles of APDs by the area of tissue in the field of view. The total ventricular surface in the field of view was defined as the whole heart. The infarct region was defined as a signal-to-noise ratio between 5 and 10 and an AP rise time (*T*_rise_) > 15 ms (27). The remote region was defined as an area of similar size but far from the infarct region and toward the base of the heart. The corresponding anatomical regions were also selected in sham hearts, with the remote area on the base and the infarct area on the left ventricular (LV) apex.

### Histology and Immunohistochemistry

Following optical mapping, six MI hearts were fixed in 4% paraformaldehyde and placed in 30% sucrose overnight. Short axis slices (∼2 mm) were cut (6 sections/heart), embedded in an optimal cutting temperature (OCT) medium, and frozen for storage at −80°C. OCT blocks were cryo-sectioned at 10 µm thickness, and each section was thaw mounted onto positively charged slides (Acepix Biosciences). Immunohistochemistry for CSPG and sympathetic nerve density with tyrosine hydroxylase (TH) was performed as previously described (7, 10, 28, 29). In brief, slides were rehydrated with phosphate-buffered saline (PBS) and incubated in sodium borohydride (10 mg/mL; 3 × 3 min). Slides were then blocked with 2% bovine serum albumin (Sigma) in PBS containing 0.3% Triton X-100 (Sigma) for 1 h and probed with primary antibodies targeting CSPG (1:300, Sigma CS-56) and TH (1:300, EMD Millipore) overnight. Secondary antibodies (goat anti-mouse and goat anti-rabbit, respectively, both 1:500) conjugated to Alexa Fluor 488 or Alexa Fluor 568 (Invitrogen) were applied to the samples for 90 min. Glycerol in PBS (1:1) was used to mount coverslips. Sections were imaged on a Nikon Eclipse Ni microscope at ×4 and ×10 magnification with a flourescein isothiocyanate (FITC) or tetramethylrhodamine isothiocyanate (TRITC) filter (Ex 470/40 or 525/50 nm). A minimum of five images were taken of the infarct and remote zone. TH labeling was quantified with Nikon NIS-Elements Basic Research Microscope Imaging Software, and the percent tissue area that was TH^+^ was calculated. To identify the location of infarct regions, Masson’s trichrome staining [Trichrome Stain (Masson) Kit, Millipore-Sigma] was performed on adjacent sections as previously described, and infarct areas identified via blue fibrosis within the LV and apical areas (10).

### Statistics

Data are expressed as means ± SD for *N* animals. Statistical analysis was performed using GraphPad Prism 9. Normality was tested using the Shapiro–Wilk test, and significance was assessed using Student’s unpaired *t* test, Mann–Whitney test, two-way ANOVA with repeated measures or mixed effects with Sidak’s multiple comparison correction, or Fisher’s exact test as appropriate and specified in the figure legend. Statistical significance was attained when *P* < 0.05.

## RESULTS

### CSPGs and Sympathetic Nerve Density

**Figure 1.**
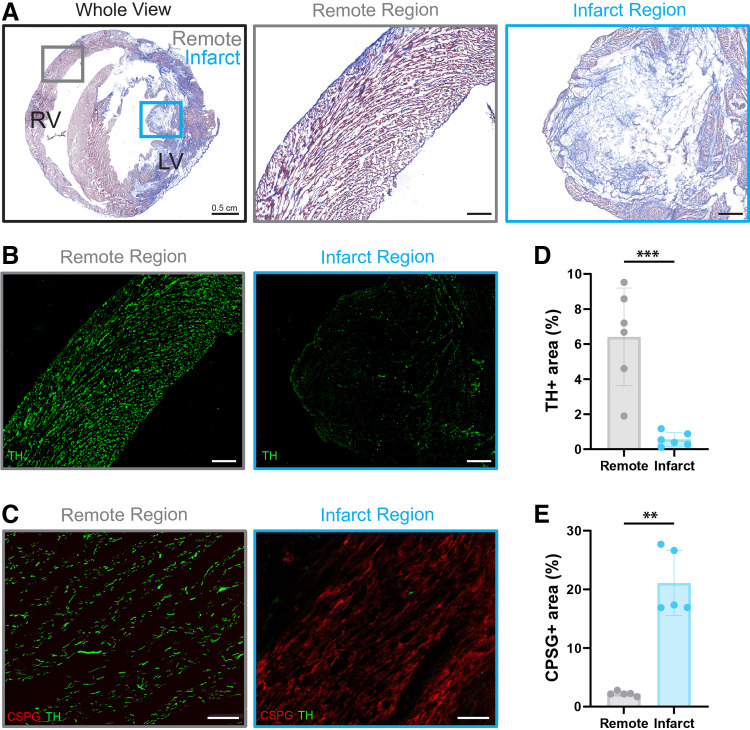
Masson’s trichrome, sympathetic nerve density, and chondroitin sulfate proteoglycans (CSPGs) in myocardial infarction (MI) hearts. *A*: Masson’s trichrome staining showing fibrosis (blue) and myocardial tissue (red) in the whole view (*left*), remote (*middle*), and infarct (*right*) regions. *B*: sympathetic nerve staining [tyrosine hydroxylase (TH); green] in the remote (*left*) and infarct (*right*) regions. *C*: CSPG (red) and TH (green) in remote (*left*) and infarct (*right*) regions. *D*: TH-positive tissue area. *E*: CSPG-positive tissue area. Data are means ± SD; *N* = 5 or 6/group. **P < 0.01 and ***P < 0.001, by unpaired *t* test (*D*) and Mann–Whitney test (*E*). Scale bars = 100 µm unless otherwise stated. TH, tyrosine hydroxylase.

Infarct presence and location were verified by Masson’s Trichrome staining (Fig. 1*A*). TH labeling revealed visible sympathetic nerve fiber loss in the infarct versus remote region (Fig. 1*B*). CSPGs were present in the infarct of all (6/6) MI hearts examined (Fig. 1*C*), with no CSPG^+^ signal observed outside the infarct area. Sympathetic nerve density was quantified and found to be reduced in the infarct versus remote region (Fig. 1*D*; 0.56 ± 0.40 vs. 6.41 ± 2.78%, *P* = 0.0005), with CSPG conversely increased in the infarct versus remote region (Fig. 1*E*; 21.1 ± 5.55 vs. 2.2 ± 0.40%, *P* = 0.008).

### Effect of SNS on Heart Rate, Electrophysiology, and Arrhythmogenesis

**Figure 2.**
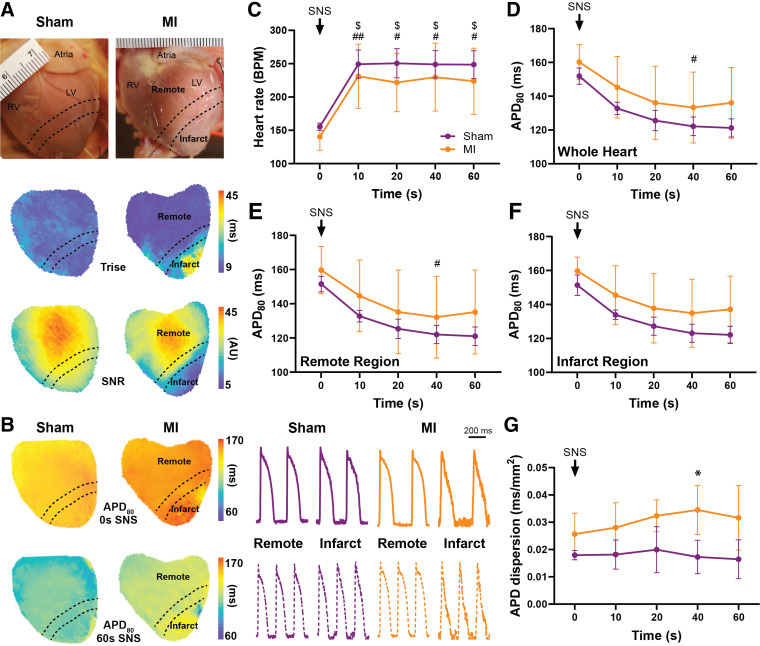
Effect of sympathetic nerve stimulation (SNS) on electrophysiological parameters during sinus rhythm. *A*: representative images and maps showing remote and infarct regions, with infarcts defined by action potential (AP) rise time (*T*_rise_) > 15 ms and signal-to-noise ratio (SNR) of <15. *B*: representative whole heart AP duration (APD) at 80% repolarization (APD_80_) maps and APs from remote and infarct regions at 0 and 60-s SNS. *C–G*: changes in heart rate (*C*), APD_80_ in the whole heart (*D*), APD_80_ in the remote region (*E*), APD_80_ in the infarct region (*F*), and APD dispersion (*G*) in response to SNS. Data are means ± SD. *C*: *N* = 4–6/group (except 20 s, *N* = 4 or 5/group). *D–F*: *N* = 3–5/group (except 60 s, *N* = 3 or 4/group). *G*: *N* = 3–5/group. *P < 0.05 vs. sham, $/#*P* < 0.05 vs. baseline in sham/myocardial infarction (MI), and ##*P* < 0.01 vs. baseline in MI, by 2-way ANOVA with mixed-effects analysis (*C–F*) and 2-way ANOVA with repeated measures (*G*).

**Figure 3.**
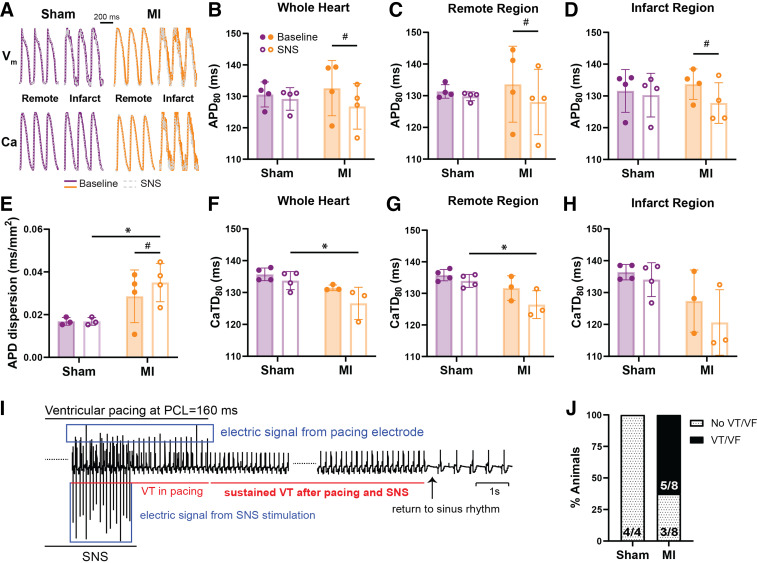
Effect of sympathetic nerve stimulation (SNS) on electrophysiological parameters and arrhythmia susceptibility during pacing. *A*: representative action potentials and Ca^2+^ transients from remote and infarct regions at pacing cycle length (PCL) = 200 ms baseline, and PCL = 200 ms with SNS. Action potential duration (APD) at 80% repolarization (APD_80_) in the whole heart (*B*), remote (*C*), and infarct (*D*) regions at PCL = 200 ms with and without SNS. *E*: APD dispersion with and without SNS. *F–H*: Ca^2+^ transient duration at 80% repolarization (CaTD_80_) in the whole heart (*F*), remote (*G*), and infarct (*H*) regions at PCL = 200 ms with and without SNS. *I*: example echocardiogram (ECG) trace of ventricular tachycardia (VT). *J*: proportion of rabbits with VT or ventricular fibrillation (VF). Data are means ± SD. *B–D*: *N* = 4/group. *E–H*: *N* = 3 or 4/group. *J*: *N* = 4–8/group. *P < 0.05 MI vs. sham, #*P* < 0.05 baseline vs. SNS in myocardial infarction (MI), by two-way ANOVA with repeated measures (*B–H*) and Fisher’s exact test (*J*).

The effect of SNS on electrophysiological parameters during sinus rhythm (Fig. 2) and pacing (Fig. 3) was assessed. During 60-s SNS in sinus rhythm, HR significantly increased by ∼100 beats/min within 10 s of stimulation in both sham and MI hearts and remained relatively stable for the remainder of stimulation (Fig. 2*C*, P = 0.02 and *P* = 0.006, respectively). APD_80_ shortened by 30.7 ± 5.8 and 24.3 ± 5.3 ms with 60-s SNS in sham and MI, respectively (*P* < 0.001), with a trend for shorter APD in sham hearts; however, no significant difference was observed between groups (Fig. 2, *D–F*). Interestingly, although APD_80_ dispersion remained constant during SNS in sham hearts, it gradually increased in MI hearts and peaked at 40 s of SNS, where APD_80_ dispersion was increased versus sham (Fig. 2*G*, P = 0.02).

To further determine the effect of SNS, rate-matched data (PCL = 200 ms) from whole heart, remote, and infarct regions with and without SNS were assessed. APD_80_ shortened in response to SNS in the whole heart, remote, and infarct regions of MI hearts; however, no significant differences were observed in sham (Fig. 3, *B–D*). Similarly, APD_80_ dispersion was unaltered following SNS in sham hearts but increased in MI (Fig. 3*E*). Although no difference in CaTD_80_ with SNS was observed versus baseline, SNS CaTD_80_ was shorter in MI versus sham in the whole heart and remote regions (Fig. 3, *F* and *G*, both *P* = 0.02).

To assess susceptibility to arrhythmia, rapid ventricular pacing at increasing rates (shorter PCLs) was performed during SNS to induce VT/VF (Fig. 3*I*). Although not significant, there was a tendency for increased arrhythmias in MI with VT/VF induced in 5/8 MI hearts versus 0/4 in sham (Fig. 3*J*, *P* = 0.08).

### β-Adrenergic Responsiveness

**Figure 4.**
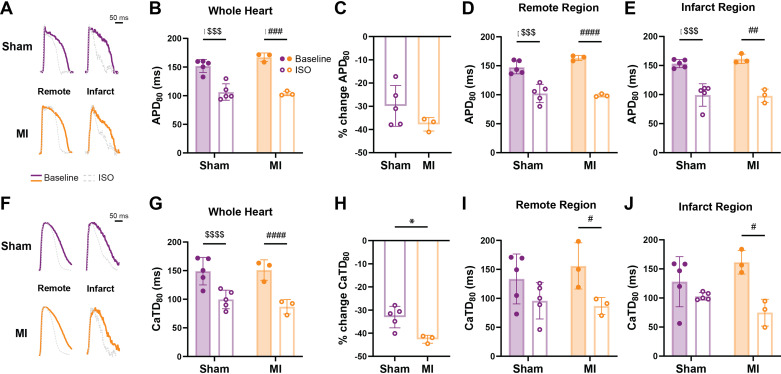
Effect of isoproterenol (Iso) on action potential duration at 80% repolarization (APD_80_) and Ca^2+^ transient duration at 80% repolarization (CaTD_80_) during sinus rhythm. *A*: representative action potentials from remote and infarct regions during sinus rhythm at baseline and with isoproterenol (Iso). *B–E*: APD_80_ (*B*) and percent change in APD_80_ (*C*) in the whole heart and APD_80_ in the remote (*D*) and infarct (*E*) regions. *F*: representative Ca^2+^ transients from remote and infarct regions during sinus rhythm at baseline and with Iso. *G–J*: CaTD_80_ (*G*) and percent change in CaTD_80_ (*H*) in the whole heart, CaTD_80_ in the remote (*I*) and infarct (*J*) regions in response to Iso. Data are means ± SD; *N* = 3–5/group. *P < 0.05 myocardial infarction (MI) vs. sham, $$$/###*P* < 0.001, $$$$/####*P* < 0.0001 baseline vs. Iso in sham/MI, by two-way ANOVA with repeated measures (*B*, *D*, *E*, *G*, *I*, and *J*) or unpaired *t* test (*C* and *H*).

As differences in electrophysiological responses to SNS may arise from altered sympathetic nerve density or function [e.g., local norepinephrine (NE) release], altered cardiomyocyte responsiveness to β-adrenergic stimulation, or a combination of both, we specifically evaluated β-adrenergic responsiveness across the ventricle using Iso. APD_80_ and CaTD_80_ shortened in sinus rhythm with Iso in both sham and MI (Fig. 4*B*, both *P* = 0.0003; and Fig. 4*G*, both *P* < 0.0001), with slightly greater relative Iso-mediated shortening of CaTD_80_ in MI versus sham (Fig. 4*H*, −42.60 ± 1.71 vs. −33.04 ± 4.67%, *P* = 0.02). These changes were reflective of changes in the remote and infarct regions (Fig. 4, *D*, *E*, *I*, and *J*).

## DISCUSSION

Here, we show that *1*) CSPGs are present in the hypoinnervated infarct region of rabbit hearts with reperfused MI, *2*) SNS increases APD dispersion and tendency for VT/VF in MI, and *3*) despite significant hypoinnervation, MI hearts remain responsive to physiological nerve stimulation, potentially via preserved or slightly elevated β-adrenergic responsiveness.

Similar to previous studies in mice (7, 11), we found CSPGs present in both the infarct and border zone regions of post-MI rabbit hearts at 10 days post-MI (Fig. 1, *C* and *E*), with the infarct region having a significant reduction in sympathetic nerve density compared with the remote region (Fig. 1, *B* and *D*). CSPGs, particularly when sulfated, mediate inhibition of nerve regrowth post-MI through binding to protein tyrosine phosphatase receptor-σ (PTPRσ) present on sympathetic neurons. Deletion, modification, or pharmacological inhibition of PTPRσ or CSPG sulfation has been shown to promote sympathetic reinnervation of the infarct (7, 11, 28, 30). Although sulfation of CSPGs was not assessed in this study, we found the presence of CSPGs to be associated with hypoinnervation, suggesting similar mechanisms of post-MI sympathetic remodeling that occur on a similar timescale between rodents and rabbits. Interestingly, as CSPGs are absent in nonreperfused MI infarcts [e.g., chronic ligation models (11)], the cellular source of CSPGs and how CSPG expression is impacted by reperfusion remains an important area for future study.

Both sham and MI hearts demonstrated similar changes in HR and APD during SNS in sinus rhythm; however, SNS resulted in a gradual increase in APD dispersion in MI but not sham hearts (Fig. 2*G*). These results are comparable to previous studies in models of MI and sympathetic denervation without MI (7, 10), in patients with postinfarct cardiomyopathy (9), and in a rabbit MI-induced heart failure model where APD restitution dispersion was increased (31). The increased APD dispersion we observed here is likely consequential of heterogeneous sympathetic innervation and potentially heterogeneous changes in β-adrenergic sensitivity post-MI. As increased APD dispersion or heterogeneity of refractoriness provides a substrate for unidirectional conduction block and re-entry (13), this likely underlies the tendency for increased susceptibility to pacing-induced ventricular arrhythmias during SNS in post-MI hearts (Fig. 3*J*) (31).

Post-MI changes in responsiveness to SNS or β-adrenergic stimulation may also contribute to the observed differences in APD_80_ and CaTD_80_. Despite reduced sympathetic nerve density in the infarct region, APD_80_ shortened in response to SNS in MI (Fig. 3, *B–D*), indicating that the hypoinnervated rabbit myocardium is still sensitive and responsive to SNS, in line with a previous study (31). These findings could be due to changes in the amount of NE released from the remaining sympathetic nerves in the infarct region, changes in NE reuptake (which may result in increased NE diffusion from nearby nerves), or changes in β-adrenergic sensitivity of the myocardium. Although Iso resulted in the shortening of APD_80_ and CaTD_80_ from baseline values in both sham and MI hearts, slightly greater relative changes in CaTD_80_ were observed in MI (Fig. 4), which may suggest enhanced responsiveness of Ca^2+^-handling proteins modulated by β-adrenergic activation (e.g., phospholamban phosphorylation on sarcoendoplasmic reticulum calcium ATPase activity). Taken together, these results suggest that at 10 ± 1 days post-MI, rabbit hearts are at least as sensitive to direct stimulation of β-adrenergic receptors as sham hearts, with perhaps slightly elevated sensitivity as indicated by the larger change in CaTD_80_. Although β-adrenergic sensitivity likely changes throughout the post-MI time course, an exaggerated sympathetic effect has also been observed following 8 wk of post-MI-induced heart failure where intrinsic cardiac ganglia in the hilum region were enlarged (31).

## CONCLUSIONS

Here, we used the innervated rabbit heart model to evaluate electrophysiological and sympathetic remodeling 10 ± 1 days following reperfused MI. We found that CSPGs were present in the infarct region and were associated with significant sympathetic hypoinnervation, suggesting that the mechanisms of post-MI sympathetic remodeling may be similar in rodents and larger mammals. Moreover, despite hypoinnervation, post-MI hearts retained sympathetic responsiveness at this post-MI timepoint while demonstrating proarrhythmic responses to SNS.

## DATA AVAILABILITY

Full data sets are available from the corresponding author upon reasonable request.

## GRANTS

This study was funded by National Institutes of Health Grants R01HL111600, R01HL093056, and T32GM144303 and National Natural Science Foundation of China Grant 82200346. G.A.N. has been supported by the British Heart Foundation Programme Grant RG/17/3/32,774 and Medical Research Council Biomedical Catalyst Developmental Pathway Funding Scheme MR/S037306/1.

## DISCLOSURES

No conflicts of interest, financial or otherwise, are declared by the authors.

Crystal Ripplinger is an editor of *American Journal of Physiology-Heart and Circulatory Physiology* and was not involved and did not have access to information regarding the peer-review process or final disposition of this article. An alternate editor oversaw the peer-review and decision-making process for this article.

## AUTHOR CONTRIBUTIONS

B.W.M., G.A.N., B.A.H., Z.W., and C.M.R. conceived and designed research; A.G., L.W., J.L.C., S.T., S.D.F.S., B.W.M., and Z.W. performed experiments; A.G., C.E.R.S., L.W., J.L.C., S.T., S.D.F.S., Z.W., and C.M.R. analyzed data; A.G., C.E.R.S., L.W., J.L.C., S.T., S.D.F.S., B.W.M., G.A.N., B.A.H., Z.W., and C.M.R. interpreted results of experiments; A.G., C.E.R.S., J.L.C., Z.W., and C.M.R. prepared figures; A.G., C.E.R.S., Z.W., and C.M.R. drafted manuscript; A.G., C.E.R.S., J.L.C., S.D.F.S., G.A.N., B.A.H., Z.W., and C.M.R. edited and revised manuscript; A.G., C.E.R.S., L.W., J.L.C., S.T., S.D.F.S., B.W.M., G.A.N., B.A.H., Z.W., and C.M.R. approved final version of manuscript.
